# On the Aging of OTFTs and Its Impact on PUFs Reliability

**DOI:** 10.3390/mi15040443

**Published:** 2024-03-26

**Authors:** Marc Porti, Gerard Palau, Albert Crespo-Yepes, August Arnal Rus, Simon Ogier, Eloi Ramon, Montserrat Nafria

**Affiliations:** 1 Department of Electronic Engineering, Universitat Autònoma de Barcelona, 08193 Bellaterra, Spain; gerard.palau.b@gmail.com (G.P.); albert.crespo.yepes@gmail.com (A.C.-Y.); montse.nafria@uab.es (M.N.); 2 FlexiIC SL, 08173 Sant Cugat del Vallés, Spain; august.arnal@flexiic.tech; 3 SmartKem Ltd., Neville Hamlin Building, Thomas Wright Way, NetPark, Sedgefield TS21 3FG, UK; s.ogier@smartkem.com; 4 Institut de Microelectrònica de Barcelona (IMB-CNM-CSIC), 08193 Bellaterra, Spain; eloi.ramon@imb-cnm.csic.es

**Keywords:** OTFT, variability, reliability, PUF, aging, BTI, HCI

## Abstract

Given the current maturity of printed technologies, Organic Thin-Film Transistors (OTFT) still show high initial variability, which can be beneficial for its exploitation in security applications. In this work, the process-related variability and aging of commercial OTFTs have been characterized to evaluate the feasibility of OTFTs-based Physical Unclonable Functions (PUFs) implementation. For our devices, *I_D_*-based PUFs show good uniformity and uniqueness. However, PUFs’ reliability could be compromised because of the observed transient and aging effects in the OTFTs, which could hinder the reproducibility of the generated fingerprints. A systematic study of the aging of OTFTs has been performed to evaluate the PUFs’ reliability. Our results suggest that the observed transient and aging effects could be mitigated so that the OTFTs-based PUFs’ reliability could be improved.

## 1. Introduction

Due to their low cost, sustainability and easy fabrication, the study of organic materials in devices as Thin-Film Transistors (TFT) has considerably increased during the last years [1,2]. The potential use of these materials in devices for the Internet of Things (IoT) and wearable electronics has been explored thanks to the possibility of fabricating smart and flexible electronic systems [3]. Due to the good characteristics of these materials, many works have been focused on the study of the performance of the devices fabricated with organic materials [4] or on the properties of the materials themselves [5,6,7]. However, not many studies have been devoted to the analysis of the process-related variability and reliability of OTFT devices [5,8,9,10]. Since the fabrication of organic materials is not a mature enough process yet, the devices show high initial variability (i.e., Time-Zero Variability, TZV). On the positive side, TZV of Organic TFTs (OTFTs) is continuously improving as the technology matures (and, consequently, their performance as well) but, also, the large performance variability of the OTFTs can be exploited for some security and cryptography applications [11]. The randomness associated to the heterogeneous nature of organic materials and the fabrication processes can be used as a source of entropy for the implementation of Physical Unclonable Functions (PUFs) [11,12,13]. However, the reliability of OTFT devices is another issue of concern due to its direct impact on the PUFs’ reliability, i.e., for the reproducibility of the cryptographic key. In this regard, the usual operating conditions of this kind of device can be a serious drawback because the required biasing could trigger aging mechanisms that progressively degrade the organic materials’ properties and the device performance, leading to a time-dependent variability (TDV). Actually, in our previous work [11], we observed that the drift of the drain current in the OTFTs during operation could end in a change in the PUF output, with a consequent reduction in the PUF reliability. Therefore, a complete evaluation of the performance/reliability of PUFs requires a detailed study of the aging mechanisms that are involved in the materials used for the fabrication of OTFTs and their impact on the electrical characteristics of these devices [10]. 

In this manuscript, the TZV of commercial OTFTs has been characterized. Considering the drain current (*I_D_*) as the entropy source, the suitability of OTFTs for PUF implementation has been evaluated by determining the homogeneity and uniqueness of the generated PUF outputs. With the purpose of evaluating the PUF reliability, an extensive study of the impact of Bias Temperature Instabilities (BTI) and Hot Carrier Injection (HCI)-like electrical tests on the OTFTs drain current has been carried out. Large transients and significant current drifts of the *I_D_* current are observed, which are strongly dependent on the applied stresses, hindering the reliability of the corresponding PUF. However, for some specific bias conditions, the effects tend towards saturation, suggesting that a properly chosen electrical pre-stress test may help to reduce/mitigate the transient/aging effects and, consequently, improve the reproducibility of PUFs.

## 2. Experimental 

**Figure 1:**
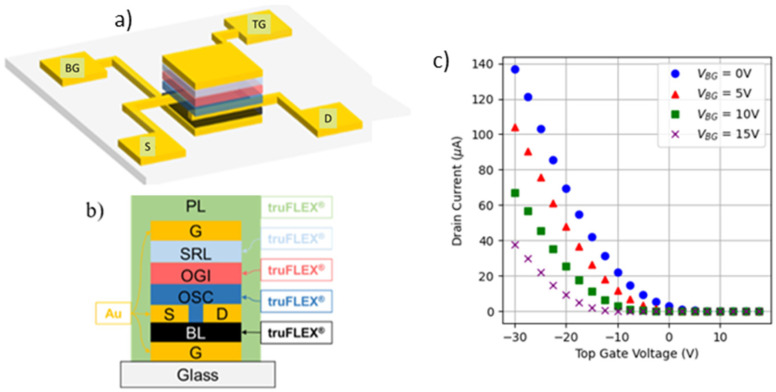
(**a**) Three-dimensional sketch of the studied OTFTs where source (*S*), drain (*D*), top gate (*TG*) and back gate (*BG*) terminals are shown and (**b**) the device cross-section. (**c**) Examples of *I_D_-V_TG_* curves obtained at different back gate voltages (*V_BG_*) on an OTFT, with *V_DS_* = −25 V.

The investigated devices are four-terminal transistors with a top gate configuration and multiple channels (Figure 1a,b) [4]. The terminals are source (*S*), drain (*D*), top gate (*TG*) and bottom gate (*BT*). The devices were fabricated by SmartKem Ltd. (Manchester, UK), using gold contacts (*G*) and their proprietary TRUFLEX stack of materials, formed by base layer (BL), organic semiconductor (OSC), organic gate insulator (OGI), sputter-resistant layer (SRL) and passivation layer (PL). The fabrication process combines sputtering, spin coating and photolithographic steps. The nominal operation voltage of these devices is in the range of ±10 V–±30 V. In this work, devices with W = 360 µm and L = 2.5 µm have been characterized. 

Figure 1c shows, as an example, typical *I_D_-V_TG_* curves measured on an OTFT at *V_DS_* = −25 V (saturation regime) for different back gate biases (*V_BG_*). Note that, as *V_BG_* increases, a shift of the curve towards negative voltages is observed, as expected, shifting the threshold voltage (*V_TH_*) to negative values. In our case, *V_BG_* = 10 V is considered in the rest of the manuscript. For the analysis of the TZV, the *I_D_-V_TG_* curves on 70 OTFTs were measured, from which the drain current at a fixed voltage is obtained, to determine the statistical dispersion of this current.

To evaluate the time-dependent variability of these devices, a typical measurement–stress–measurement (MSM) scheme has been adopted. Bias temperature instabilities (BTI) and hot carrier injection (HCI)-like tests have been applied at different voltages and times. For the BTI-like tests, a voltage was applied to the top gate (*V_TG_*) with drain (*V_D_*) and source (*V_S_*) grounded, while, for the HCI-like tests, top gate and drain voltages were simultaneously applied (with *V_TG_ = V_D_*) with the source grounded. Positive and negative voltages were applied. In particular, BTI and HCI-like tests at ±10, ±20, ±30 V for 600 s have been considered. Note that these voltages are within the nominal operation range of our devices, differently to the commonly used accelerated conditions in reliability tests. However, since an MSM methodology has been adopted, to clearly distinguish between the voltages applied during the stress phases from those during the measurement ones, we will refer to them as “stress voltages”. In some devices, the impact of the stress was only evaluated at the end of the test. In some others, the stress was interrupted to measure the progressive change in device properties. This was accomplished by applying *V_TG_* = −10 V and V_D_ = −10 V during 100 s in the measurement phase. Transient effects are also analyzed by measuring the *I_D_-t* curves under given *V_TG_* and *V_D_* during both the stress and measurement phases. Of course, the time-dependent variability of OTFTs do not only depend on the stress suffered during operation conditions but on other parameters as environmental effects and temperature. In our case, since the devices are protected by the double gate structure, environmental effects are not relevant. However, temperature effects should also be considered in future studies. In our case, all measurements were performed at room temperature. 

## 3. Results

### 3.1. OTFTs Time-Zero Variability and PUF Performance

Large time-zero variability of OTFT devices has been reported in the literature and has been considered as a drawback of this technology that must be overcome for its commercial use. However, many cryptography-based systems and applications take advantage of these random phenomena. Therefore, the large TZV variability of these devices could be useful for PUF applications. In this work, we have considered a PUF that uses the current through the channel (*I_D_*) of the device as the source of entropy, i.e., the drain current is the challenge from which the PUF provides a key as response. As a mapping function for key generation, the digitalization of the *I_D_* current into a given number of bits is considered [11]. Keys are generated by concatenating the words provided by several OTFTs. To evaluate the suitability of the OTFTs for key generation using this procedure, the homogeneity, uniqueness, and reliability of the implemented PUF outcomes must be evaluated. 

**Figure 2:**
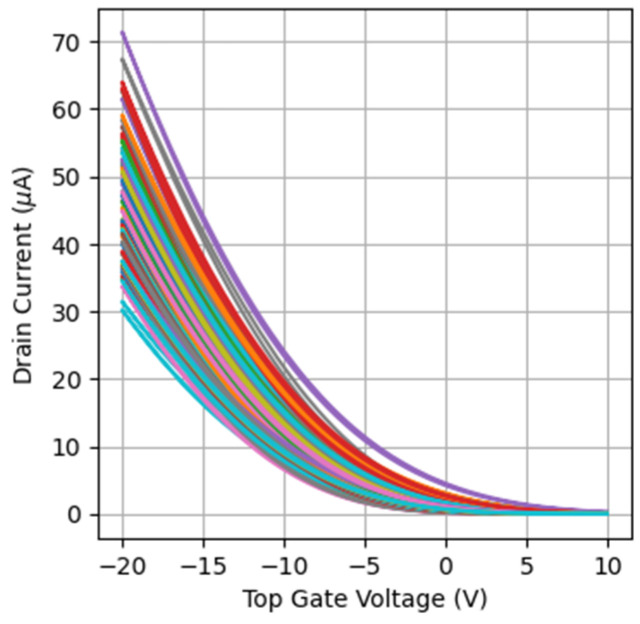
*I_D_-V_TG_* curves measured on a set of OTFTs at *V_DS_* = −15 V, *V_BG_* = 10 V (L = 2.5 µm and W = 360 µm). A large dispersion is observed. *I_D_* is given in absolute value.

First of all, the statistical distribution of the *I_D_* current in a set of devices has been evaluated. Figure 2 shows the *I_D_-V_TG_* (*I_D_* is given in absolute value in the rest of the manuscript) curves measured on 70 nominally identical devices at *V_DS_* = −15 V and *V_BG_* = 10 V (saturation regime). Note that a high device-to-device variability is observed. Since, for the evaluation of the PUF quality, a large number of devices is needed (larger than that experimentally available), many OTFT *I_D_-V_TG_* curves have been randomly generated based on the experimental data. To achieve this, the threshold voltage (*V_TH_*) and mobility (*µ*) of each of the measured devices have been determined by fitting the experimental *I_D_-V_TG_* curves (Figure 2) to Equation (1):

(1) $$I_{D}=\frac{W}{L}\mu C_{d}\frac{1}{2}{\left(V_{TG}-V_{TH}\right)}^{2}$$

**Figure 3:**
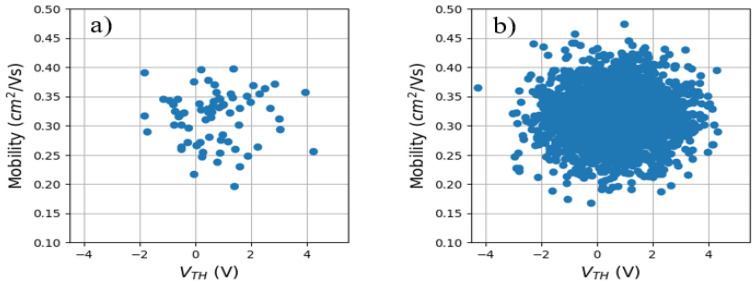
(**a**) Mobility vs. threshold voltage of the measured devices. (**b**) A total of 2400 mobility-threshold voltage pairs have been randomly generated to increase the size of the *I_D_-V_TG_* set.

To obtain *V_TH_* and *µ* of all the devices, we have followed the following procedure. Their *I_D_-V_TG_* curve has been linearized by plotting the square root of *I_D_* vs. *V_TG_.* Then, the slope is proportional to the mobility of the device and the intersection with the X-axis provides the threshold voltage. Figure 3a shows the *µ* vs. *V_TH_* plot obtained from the experimental curves. Note that there is no correlation between the extracted parameters. Moreover, TZV is quite high, which can be very useful for PUF implementation. In our case, an average value and dispersion of 0.71 ± 1.25 V and 0.31 ± 0.04 cm^2^/Vs were found for *V_TH_* and *µ*, respectively.

Once the experimental statistical distributions of µ and *V_TH_* have been determined, a set of data with 2400 *V_TH_*–*µ* pairs (i.e., OTFTs) have been randomly generated using the statistics obtained experimentally. Its average and dispersion have been obtained, being *V_TH_* = 0.73 ± 1.30 V and *µ* = 0.31 ± 0.04 cm^2^/Vs. Note that the experimental and generated data show similar statistical values. From the set of generated *V_TH_–µ* pairs, the *I_D_* of OTFTs at *V_GS_* = −10 V and *V_DS_* = −15 V has been obtained by using Equation (1). Two cases were considered. In the first case, the I_D_ of a sub-set of 1200 devices was digitized into 8 bits; in the second case, the *I_D_* of 2400 devices were binarized into 4-bit word outcomes. From these 8-bit or 4-bit binary words, cryptographic keys of 32 bits have been generated by concatenating the digitized currents of 4 or 8 different devices, respectively. Using this methodology, 300 PUFs with 32-bit outcomes were built for the two cases. 

The uniformity and uniqueness of the proposed PUFs have been determined to evaluate the quality of the generated fingerprints. The bit uniformity, which is a measure of the random distribution of “0s” and “1s”, is assessed first. The uniformity of a PUF is determined by dividing the number of 0 bits by the total number of bits of the corresponding key, as given by Equation (2):

(2) $$PUF\;Uniformity=\frac{1}{s}\sum_{i=1}^{s}K_{i}\times 100\%$$

where *s* is the key size and *K_i_* the bit value at location *i* in the *PUF*. In our case, the uniformity mean value ($\bar{X})$ and standard deviation (*SD*) for the PUFs based on devices whose *I_D_* was digitized into 8-bit or 4-bit binary words is 0.50 ± 0.07 and 0.52 ± 0.06, respectively (see Table 1). Note that the average values are very close to 0.5, which is the expected value for the ideal situation. To evaluate the degree of correlation between the binary keys of two different PUFs, the device uniqueness must be assessed. The device uniqueness is determined from the inter-device Hamming Distance (HD), which measures the number of bits that are different with respect to the total number of bits of the key when the keys of two different PUFs are compared. The inter-device HD between any two PUFs can be calculated with Equation (3):

(3) $$Device\;uniqueness=\frac{2}{q(q-1)}\sum_{i=1}^{q-1}\sum_{j=i+1}^{q}\frac{HD(K_{i},K_{j})}{s}\times 100\%$$

where *K_i_* and *K_j_* are s-bit keys of the *i*th *PUF* device and the *j*th *PUF* device among *q* different PUFs, respectively. In our case, since we have generated keys for 300 PUFs, a total number of 300 × 299/2 = 44,850 combinations are possible. The average value ($\bar{X})$ and *SD* for the uniqueness is 0.51 ± 0.09 for the currents digitized into 8 bits and 0.54 ± 0.10 for the currents digitized into 4 bits (see Table 1). These values are, again, very close to the 0.5 ideal case.

### 3.2. Impact of Electrical Stresses on the OTFTs’ Performance

As for the reliability of the PUFs, i.e., the reproducibility of the generated fingerprints, our previous work [11] showed that, considering device aging during an HCI-like stress, the associated shift of the device current could be a drawback for this kind of application. However, those preliminary results also showed that the current shift saturates with the stress time, suggesting that the application of a carefully chosen stress previously to the PUF operation may be beneficial for its reliability. In this work, an extensive characterization of the changes in OTFTs’ electrical characteristics when subjected to electrical stress has been carried out. Since the considered PUFs are based on the digitalization of the *I_D_* current, which may be affected by aging mechanisms during the device operation, in this work, an extensive characterization of this aging has been performed on our devices. 

**Figure 4:**
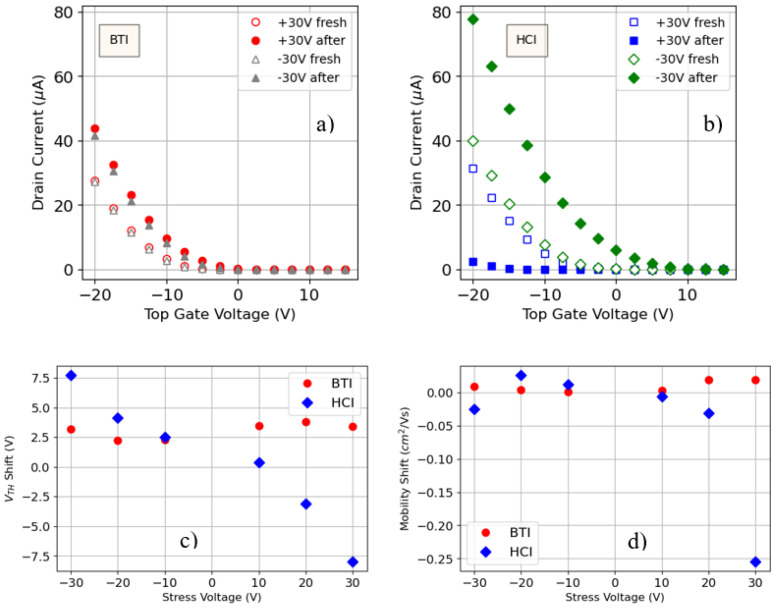
Examples of *I_D_-V_TG_* curves before and after BTI (**a**) and HCI-like (**b**) stresses at ±30 V. *V_TH_* (**c**) and *µ* (**d**) variation for the different stresses. Red circles and blue diamonds correspond to BTI and HCI-like tests, respectively.

To begin with, the aging of the OTFTs during BTI and HCI-like stress (see Section 2 for details) has been be evaluated by comparing the *I_D_-V_TG_* curves of the devices before and after a noninterrupted 600 s long test. Figure 4 shows, as typical examples, the *I_D_-V_TG_* curves before and after the BTI (a) and HCI-like (b) stresses at ±30 V. Note that, after the stress, the electrical characteristics have changed, demonstrating an aging effect. This change strongly depends on the stress type (BTI or HCI) and also on the value and polarity of the stress voltage. For example, whereas, for BTI, the curves are always shifted towards the left (what is indicative of an increase, in absolute value, of the threshold voltage), for HCI, the curve can shift towards the left or towards the right. This stress dependence has been quantitatively analyzed from the evaluation of the *V_TH_* and *µ* shifts experienced by the different devices. *V_TH_* and *µ* at the end of the stress have been computed as described in Section 3.1 for the fresh devices. For a given device, the shift has been calculated by comparing *V_TH_* and µ after the stress with their values obtained before the stress for that device. The results are plotted in Figure 4c,d, which show, respectively, the *V_TH_* shift and the variation in *µ* for the different analyzed cases as a function of the stress voltage. Note that, for BTI, the *V_TH_* shift is always positive, i.e., *V_TH_* increases in absolute value (unlike for MOSFET technologies) and does not show a significant dependence on the stress voltage. However, for HCI, there is a clear voltage dependence of the *V_TH_* shift, being positive/negative for negative/positive stress biases and increasing with the absolute value of the stress voltage. In the case of *µ*, independently of the stress type, no clear dependence on the stress voltage is observed for negative voltages. However, for positive voltages, it seems to increase (in absolute value) and, in the case of +30 V HCI test, a much large deviation is measured. In any case, note that the stresses have changed the electrical properties of the devices, which could be detrimental for PUF applications. 

**Figure 5:**
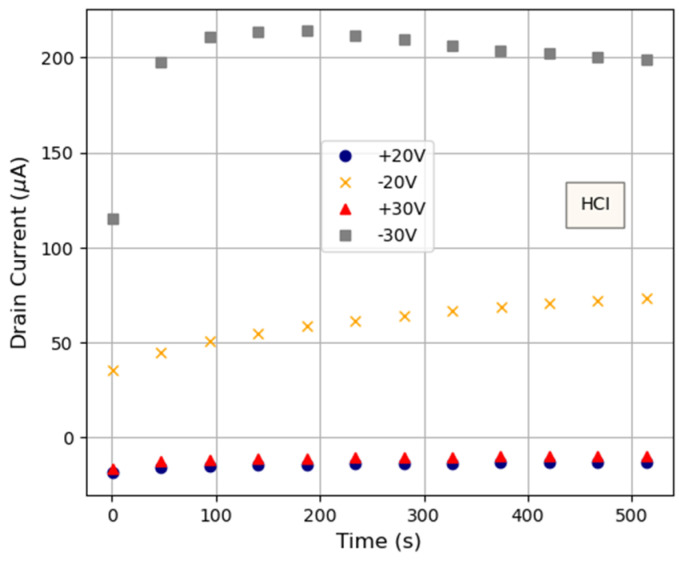
*I_D_-t* curves measured during some of the 600 s stress tests. A different (fresh) device was measured for each of the stress conditions.

The time evolution of *I_D_* during electrical stresses has been studied. Figure 5 shows the *I_D_-t* curves registered during several of the 600 s HCI-like stresses. Interestingly, in all cases, *I_D_* changes with time. In most of the cases, a fast initial increase is measured, while the current tends to saturate for larger stress times. However, the observed behavior for the −30 V HCI stress is somehow different, since, though the initial transient is also observed (very large in this case), from *t*~150 s, the current slightly decreases until a stable value seems to be reached. Therefore, two phenomena seem to play an important role in this case: the effects of the stress itself, which would be responsible for the final decrease in the current, and some kind of transient effects, responsible for the initial current increase. 

**Figure 6:**
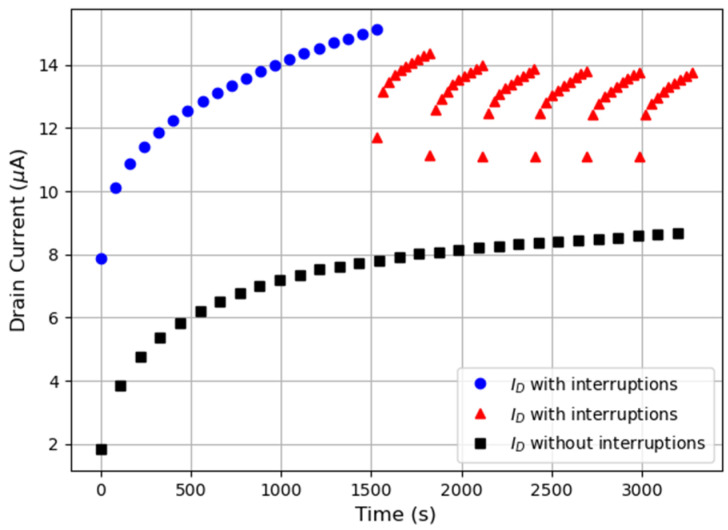
Time evolution of *I_D_* measured on one OTFT when biased under *V_TG_* = *V_D_* = −10 V during 3600 s without interruptions (black squares) and with interruptions (blue circles and red triangles). In this second case, during the first 1600 s, the bias was applied without interruptions and, afterwards, 6 cycles of 30 s interruptions + 320 s bias were applied.

The observation of a current evolution during biasing (for all the evaluated voltage conditions) suggests that the key generated by the PUF depends on the time at which it is generated, hindering the PUF reliability. Therefore, to analyze this issue, a more detailed study of current evolution has been carried out. Figure 6 shows a typical evolution of *I_D_* in two different OTFTs during an HCI test at *V_D_* = *V_TG_* = −10 V, without and with biasing interruptions. In the first case, without biasing interruption (black squares), the current was continuously measured during 1 h. In the second device (with bias interruptions), the bias was first applied during 1600 s (blue circles in the figure) and, afterwards, the bias was interrupted during 30 s and resumed for 320 s, in a six-cycle sequence (in red triangles). It is important to emphasize that both the currents plotted in blue circles and red triangles are measured on the same OTFT. Note that similar current transients are observed at the beginning of the test in both cases, but, interestingly, bias interruption strongly affects the subsequent current evolution. Lower currents are measured after the bias is resumed and current transients are also observed, which imply a current increase. However, despite the current increase during these transients, the current does not reach the values measured before the bias interruption. The observed current relaxation and transient behavior could be linked to trapping/detrapping of charges in the device when removing/resuming the bias, similarly as in BTI tests in MOSFETs [14]. From the PUF perspective, because of the large relaxation of the current after the interruptions and the presence of current transients, the measured current could be strongly dependent on the time elapsed since the bias removal (i.e., since the previous key generation of the PUF). Since the current shift can be large enough to drive *I_D_* to other binarized levels, failure of the PUF functionality (i.e., different generated codes) could occur.

However, it is important to emphasize that, as can be observed in Figure 5 and Figure 6, the initial transients seem to disappear for long enough times, reaching more stable values of *I_D_*, and this would be very beneficial for PUF implementation with OTFTs. Actually, for PUFs implemented in other technologies, to increase their reproducibility, the application of electrical stresses that induce aging effects that change permanently the device or circuit characteristics has been proposed [15,16]. So, in the next section, the suitability of this approach for the improvement of the reliability of the PUFs implemented with our devices has been explored.

## 4. Pre-Stress as a Way to Improve PUFs’ Reliability

**Figure 7:**
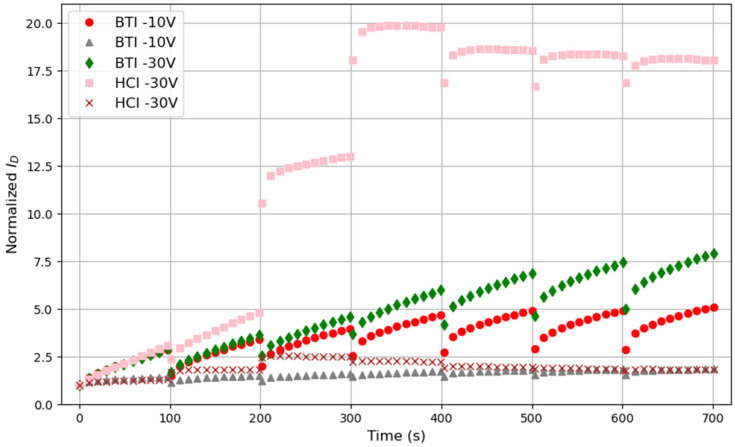
Time evolution of *I_D_* registered during the measurement phases on the fresh device (from 0 to 100 s) and after the 6 stress cycles on different devices. For each case, the current has been normalized to the initial current of the fresh device at *t* = 0.

**Figure 8:**
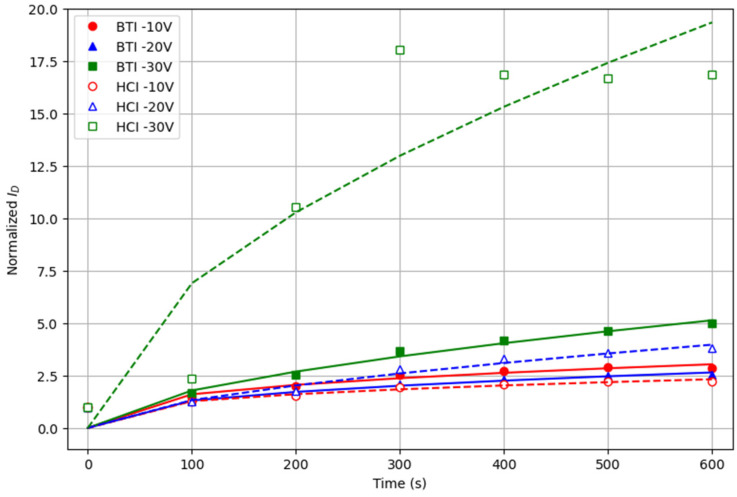
Initial currents registered during the measurement phases on different devices subjected to different stresses. To allow the plot using the same scale, all currents have been normalized to the current measured in the fresh device.

To evaluate the aging of OTFTs under biasing conditions closer to those of PUF operation (i.e., key reading + idle periods, during which the PUF is biased and unbiased, respectively), the traditional measurement–stress–measurement (MSM) scheme has been adopted. BTI and HCI-like stresses of −10 V, −20 V and −30 V have been applied to different devices, which have been periodically interrupted to measure *I_D_-t* curves, from which the impact of the stress on the device conduction, *I_D_*, has been be evaluated. In particular, six stress cycles (the first one 10 s long, the second one 100 s long and the rest 400 s long) were applied to each device. Before and after each stress cycle, the *I_D_-t* curves were measured at *V_D_* = *V_TG_* = −10 V during 100 s. *I_D_-V_TG_* curves were measured before and after the complete MSM sequence. Figure 7 shows, as examples, the *I_D_-t* curves measured (at *V_TG_* = *V_D_* = −10 V) before the first stress and after each of the stress cycles for the −10 V and −30 V BTI and HCI-like stress on different devices. In this plot, the *I_D_-t* curve in the time interval from 0 to 100 s is measured on the fresh device, while the subsequent ones correspond to those measured after each of the stress phases of the test. To allow the representation of the curves using the same vertical scale, the current has been normalized for each device to the initial current measured before the stress. Each stress condition was applied to a different fresh device. The curves corresponding to two different devices are displayed for the cases of −10 V BTI and −30 V HCI. The transient and recovery effects described in the previous section are also observed here, independently of the type of stress and stress voltage. However, the trend can slightly differ, depending on the particular test. For example, for BTI stresses, *I_D_* during each of the measurement phases seems to continuously increase (at least in the six stress cycles shown here), whereas, for the −30 V HCI-like stresses, *I_D_* rapidly increases at the beginning until it reaches a maximum value from which it starts to slightly decrease. Finally, it is important to note that there is still variability between devices after the stress, i.e., two devices stressed under the same stress conditions show different *I_D_* (see, for example, the *I_D_-t* curves in Figure 7 for the two devices stressed under −10 V NBTI or −30 V HCI), which is very important for the PUFs’ implementation. Despite the transients observed after the stress interruption, the overall *I_D_* current evolution during the whole test increases, showing a trend towards saturation, reaching quite similar values at the beginning/end of each of the measurement phases. To evaluate the stability of *I_D_* after each stress cycle, the initial and final currents during the measurement phase have been considered as parameters. Figure 8 shows, as an example, the initial currents normalized to the current obtained in the fresh device at *t* = 0 (i.e., before any stress). Each symbol corresponds to a different device. For the final currents (not shown), a similar tendency is observed. In all cases, the curves have been fitted to a potential law (continuous line), which serves as a guide to the eye.

Note that, in some cases, as for BTI −30 V or HCI −20 V, in the considered duration of the test, the initial currents show a continuous increasing trend but, in others, as for BTI −10 V or HCI −10 V, the current tends to remain almost constant after some stress time. For example, after the fifth stress cycle, the application of the electrical stress does not imply a significant change of the conduction of the device. Therefore, the results indicate that, after a given number of stress cycles (which depends on the stress conditions), the conduction through the device tends to saturate. This opens the possibility of using such prestressed devices to implement more reliable PUFs based on the *I_D_*. For example, a five-cycle BTI −10 V or HCI −10 V stress (or some equivalent continuous stress) could be applied to the devices to reach a stable *I_D_*, after which the PUF could be operated with improved reliability. Note, however, that the homogeneity and uniqueness of these prestressed PUF should be evaluated again, as well as the final reliability. 

## 5. Conclusions

In this work, it has been demonstrated that the large TZV of OTFTs can be exploited for the implementation of PUFs based on the binarization of the I_D_ current. The implemented PUFs show good uniformity and uniqueness. However, since the I_D_ current shows transients and/or aging effects during the device operation, the PUFs’ reliability may be compromised. To further evaluate this issue, an extensive characterization of the impact of electrical stresses on the performance of the OTFTs has been performed. In particular, the impact of Bias Temperature Instabilities (BTI) and Hot Carrier Injection (HCI)-like electrical stresses on the OTFT drain current has been evaluated. Transients and current relaxation are observed when the stress is interrupted, which could be detrimental for PUF applications, since the current used by the PUF for key generation will depend on the time at which it is measured, hindering the PUF reliability. The evaluation of the drain current evolution after different kinds of stresses indicates that, after a certain stress time (which depends on the stress conditions), the conduction through the device tends to saturate. This result suggests that a properly chosen electrical pre-stress may help to reduce/mitigate the transient/aging effects and, consequently, improve the reproducibility of PUFs.

## Figures and Tables

**Table 1:** PUFs (with 32 bits), obtained from a set of 1200 (left) and 2400 (right) OTFTs, whose *I_D_* was digitized into 8-bit or 4-bit binary words, respectively.

	300 PUFs Based on I_D_ Digitized into 8-Bit Binary Words (1200 OTFTs)		300 PUFs Based on I_D_ Digitized into 4-Bit Binary Words (2400 OTFTs)	
	Unif.	Uniq.	Unif.	Uniq
$\bar{X}$	0.50	0.51	0.52	0.54
*SD*	0.07	0.09	0.06	0.10

## Data Availability

The data presented in this study are available under reasonable request from the corresponding author.
